# Co-Delivery of Paclitaxel Prodrug, Gemcitabine and Porphine by Micelles for Pancreatic Cancer Treatment via Chemo-Photodynamic Combination Therapy

**DOI:** 10.3390/pharmaceutics14112280

**Published:** 2022-10-25

**Authors:** Qiwei Wu, Xiaodong Ma, Wenhui Zhou, Rong Yu, Jessica M. Rosenholm, Weizhong Tian, Lirong Zhang, Dongqing Wang, Hongbo Zhang

**Affiliations:** 1 Department of Radiology, Affiliated Hospital of Jiangsu University, Zhenjiang 212001, China; 2 Department of Medical Imaging, Affiliated Taizhou People’s Hospital of Nanjing Medical University, Taizhou 225300, China; 3 Pharmaceutical Sciences Laboratory, Faculty of Science and Engineering, Åbo Akademi University, 20520 Turku, Finland; 4 Turku Bioscience Centre, University of Turku and Åbo Akademi University, 20520 Turku, Finland; 5 Southern Medical University Affiliated Fengxian Hospital, Shanghai 201499, China

**Keywords:** photodynamic therapy, prodrug, combination therapy, pancreatic carcinoma

## Abstract

Pancreatic carcinoma is an aggressive subtype of cancer with poor prognosis, known for its refractory nature. To address this challenge, we have established a stable nanoplatform that combines chemotherapy with photodynamic therapy (PDT) to achieve better curative efficacy. First, we designed and synthesized a disulfide-bonded paclitaxel (PTX)-based prodrug, which was further mixed with gemcitabine (GEM) and photosensitizer THPP in an optimized ratio. Subsequently, the mixture was added dropwise into amphiphilic polymer DSPE-PEG water solution to form micelles composed of DSPE-PEG nanoparticles (TPG NPs). The TPG NPs were around 135 nm, and showed great ability of DTT stimulated release of PTX and GEM. Moreover, the TPG NPs can be efficiently uptaken by pancreatic cancer PANC-1 cells and effectively kill them, especially when combined with 650 nm laser irradiation. Finally, the TPG NPs have shown enhanced long-term circulation ability and also exhibited efficient anti-tumor activity in combination with 650 nm laser irradiation in a pancreatic cancer mouse model. In summary, the designed TPG NPs possesses great potential for co-delivery of paclitaxel prodrug, GEM and THPP, which enables combined chemo-photodynamic therapy for cancer treatment. In addition, the stimulated release of PTX prodrug and GEM also allows for better targeting of tumor cells and the increased therapeutic effect against cancer cells. Overall, the TPG NPs can serve as a good candidate for pancreatic cancer treatment.

## 1. Introduction

As the most common type of pancreatic cancer, pancreatic ductal adenocarcinoma (PDAC) is highly lethal due to its highly aggressive nature and resistance to conventional treatment [1]. Surgical resection remains the preferred treatment option for PDAC, but due to the difficulty of early diagnosis of PDAC, most patients are already at an advanced stage of PDAC and it is difficult for them to undergo surgical treatment [2]. Even patients with early resection could ultimately die of tumor recurrence [3]. Therefore, international guidelines recommend adjuvant chemotherapy to improve the survival rate of patients [4]. Additionally, combined therapy has proven to be extremely promising in cancer treatment.

Gemcitabine (GEM) is the cornerstone of chemotherapy for advanced PDAC; however, due to the inherent resistance mechanism of PDAC, we have to keep exploring new treatment options [5,6]. On this basis, clinical studies have found that the median survival time of pancreatic cancer patients receiving paclitaxel (PTX) in combination with GEM was improved by about 2 months compared with the GEM group [7]. Nevertheless, despite the increased efficacy, the combination of PTX and GEM has also demonstrated stronger toxic side effects [8]. To cope with this problem, nanotechnology has been used for passive drug delivery and to reduce drug side effects. Thereby, linking PTX to albumin nanoparticles provided better pharmacokinetics [9]. In addition, lipid-coated mesoporous silica nanoparticles were also used to co-deliver PTX with GEM in pancreatic cancer mice, demonstrating low toxicity and high efficacy [10]. Furthermore, the concept of prodrugs has been further proposed on the basis of nanomedicine to target cancer cells and mitigate damage to normal tissue [11,12].

Prodrugs are chemical molecules with little or no pharmacological activity linked by covalent bonds that need to be stimulated in vivo by enzymatic or chemical reactions to be converted into functional parent drugs [13,14]. In this regard, disulfide bonds are widely used as linking bonds because of their sensitivity and corresponding responsiveness to the different redox environments in organisms [15]. The disulfide-linked nanoparticles receive a stimulus from the reducing environment within the tumor cells to achieve rapid intracellular release for improved drug bioavailability [16]. Prodrugs have been widely considered for drug design [17]; GemRA (GEM coupled with retinoic acid), a retinoid prodrug of GEM, has been validated to be suitable for pancreatic cancer treatment, which has improved the bioavailability and reduced the toxicity to normal tissues [18]. Meanwhile, the prodrug can be released at specific sites by specific external stimuli such as light, heat and ultrasound [19], while photodynamic therapy (PDT) can make use of the photodynamic effect to activate the photosensitive drugs selectively concentrated in the tumor tissue, and trigger photochemical reactions to destroy the tumor. This makes prodrugs an excellent strategy to fight tumors in tandem with PDT.

PDT has become a popular primary physical therapy for cancer treatment by activating photosensitizers positioned at the tumor site under laser light, which transfer energy to surrounding oxygen molecules to produce reactive oxygen species that ablate the cancer cells [20,21]. PDT contains three basic elements: the photosensitizer, the oxygen molecules, and the excitation light. Red or near infrared light is the main additional light source for PDT due to its high tissue penetration and indirect destruction of chemical bonds, which are the driving force behind the controlled drug release [22,23]. However, the photosensitizers can be taken up non-specifically by both tumor tissue and normal tissue, making the phototoxicity of PDT damaging to normal tissue [24,25]. For this reason, altering the distribution of the photosensitizers between the tumor site and the normal tissue so that the photothermal agents are concentrated at the tumor site can enhance the specific damage to the tumor.

Here, we have designed a disulfide-bonded PTX-based prodrug to be loaded in to DSPE-PEG nanoparticles with GEM and THPP, which provide PDT, in the suitable proportions, to enable the effective and targeted treatment of pancreatic cancer.

## 2. Materials and Methods

### 2.1. Materials

Paclitaxel (PTX), gemcitabine (GEM), 3, 3′-Dithiodipropionic acid (DTDP), 4-dimethylaminopyridine (DMAP), and N-(3-dimethylaminopropyl)-N-ethylcarbodiimide hydrochloride (EDCI) were obtained from Tansoole (Hangzhou, China). DL-Dithiothreitol (DTT), 40, 60-diamidine-20-phenylindole (DAPI) and dichloromethane (DCM) were purchased from Sigma-Aldrich (Espoo, Finland).

### 2.2. Cell Lines and Cell Culture

PANC-1 and MCF-10A cell lines were obtained from American Type Culture Collection (ATCC, Manassas, VA, USA) and cultured in a constant humidity incubator containing 5% CO_2_ at 37 °C. The PANC-1 human pancreatic cancer cell line was cultured in DMEM medium (LONZA) supplemented with 10% fetal bovine serum (FBS), penicillin (100 U/mL) and streptomycin (0.1 mg/mL). The MCF-10A cell line was cultured in DMEM/F-12 (1:1) medium (LONZA) supplemented with 10% horse serum, 20 ng/mL epidermal growth factor, 0.5 μg/mL hydrocortisone, 10 μg/mL insulin, 1% NEAA, penicillin (100 U/mL) and streptomycin (0.1 mg/mL). The Panc02 mouse pancreatic cancer cell line was purchased from the Cell Bank of the Chinese Academy of Sciences (Shanghai, China) and cultured in the Roswell Park Memorial Institute (RMPI), supplemented with 10% FBS, penicillin (100 U/mL) and streptomycin (0.1 mg/mL).

### 2.3. TPG Nanoparticles Preparation and In Vitro Release of PTX

GEM-HCl and twice the number of moles of triethylamine were stirred overnight in DMF to get the free Gen. Subsequently, GEM, PTXSSPTX, THPP, and DSPE-PEG2000 were dissolved in an ethanol solution and stirred for 20 min. The organic solvent was removed under reduced pressure using a rotary evaporator. The drug-loaded membranes were hydrated for 10 min at 37 °C using DI water to prepare TPG micelles. 0.5% Tween 80 in PBS containing 10 mM DTT was used to simulate the in vivo environment for drug release experiments.

### 2.4. ROS Generation Ability Assay

The PANC-1 cells were seeded in a glass-bottom 12-well plate (5 × 10^3^ cells per well) and cultured at 37 °C, 5% CO_2_ overnight. The cells were then exposed to different conditions (TPG NPs, TPG NPs + laser) for 48 h. After that, 0.4 W/cm^2^ of 650 nm laser was added for 5 min, and 10 μM of reactive oxygen test dye DCFH-DA were further added and incubated for 20 more min. Finally, Hochest33342 was used to stain the cell nuclei and imaged by confocal microscope.

### 2.5. Cellular Uptake Assay

The TPG NPs uptake efficiency of PANC-1 cells was detected by fluorescence microscopy and flow cytometry. PANC-1 cells were seeded in glass-bottom 6-well plates (2 × 10^5^ cells per well) and cultured at 37 °C, 5% CO_2_ overnight. The cell supernatant was then replaced with medium containing TPG NPs for 2, 4 and 6 h. After that, the cell medium was discarded, PANC-1 cells were washed with PBS 3 times and fixed with 4% paraformaldehyde, followed by adding DAPI to stain nuclei for 5 min. Representative fluorescence images of PANC-1 cells incubated with TPG NPs for 2–6 h were taken by confocal microscope. Fluorescence intensity of TPG NPs uptake by PANC-1 cells was measured by flow cytometer.

### 2.6. Cellular Lysosomal Escape Assay

A confocal microscope was used to observe TPG NPs lysosomal escape in PANC-1 cells. PANC-1 cells were firstly seeded in glass-bottom 6-well plates (2 × 10^5^ cells per well) and cultured at 37 °C, 5% CO_2_ overnight. They were then treated with TPG NPs or TPG NPs + laser for 2 h and 4 h. After that, the supernatant was replaced with medium containing Lysosome Tracker for 3 h. And then PANC-1 cells were fixed with 4% paraformaldehyde and stained nuclei with DAPI. Finally, representative fluorescence images of PANC-1 cells were taken by confocal microscope.

### 2.7. In Vivo Anti-Tumor Therapy

All animal studies were in accordance with the Animal Research Committee of Jiangsu University, China (SCXK (Jiangsu)-2018-0012). Panc02 cells (5 × 10^3^) in 50 μL PBS were injected into the right dorsal subcutis of 6-week-old C57BL/6 mice. When the tumor volume reached about 50 mm^3^, we randomly divided the mice into four groups (*n* = 6): (1) PBS (control, 100 μL); (2) PTX (2.5 mg/kg) + GEM (2.5 mg/kg); (3) TPG NPs (equal to 2.5 mg/kg PTX); and (4) TPG NPs + laser (650 nm, 0.5 W/cm^2^, 5 min). All the preparations were injected through tail veins, and the laser group was irradiated with laser on the second day after drug administration. Treatments were performed once every two days through the tail vein for 16 days, and the laser group received laser treatment for 5 min the next day. During the treatment, tumor volume and mouse body weight were measured and recorded. After completion of the experiment, the tumor tissues and the major organs (heart, liver, spleen, lung and kidney) were removed from the mice for further H&E staining. Representative images of tissues were taken by a pathological section scanner (*n* ≥ 6).

### 2.8. In Vivo Fluorescence Imaging

To determine the biodistribution of TPG NPs in vivo, tumor-bearing C57BL/6 mice were treated with tail vein injections of THPP and TPG NPs, respectively, when the tumor volume reached 100 mm^3^. Images were acquired by an IVIS Lumina imaging system (Capiler, Hopkinton, MA, USA) at 6 h, 24 h and 48 h time points, respectively.

## 3. Results

### 3.1. PTX-SS-PTX, TPG NPs Synthesis and Characterization

**Figure 1:**
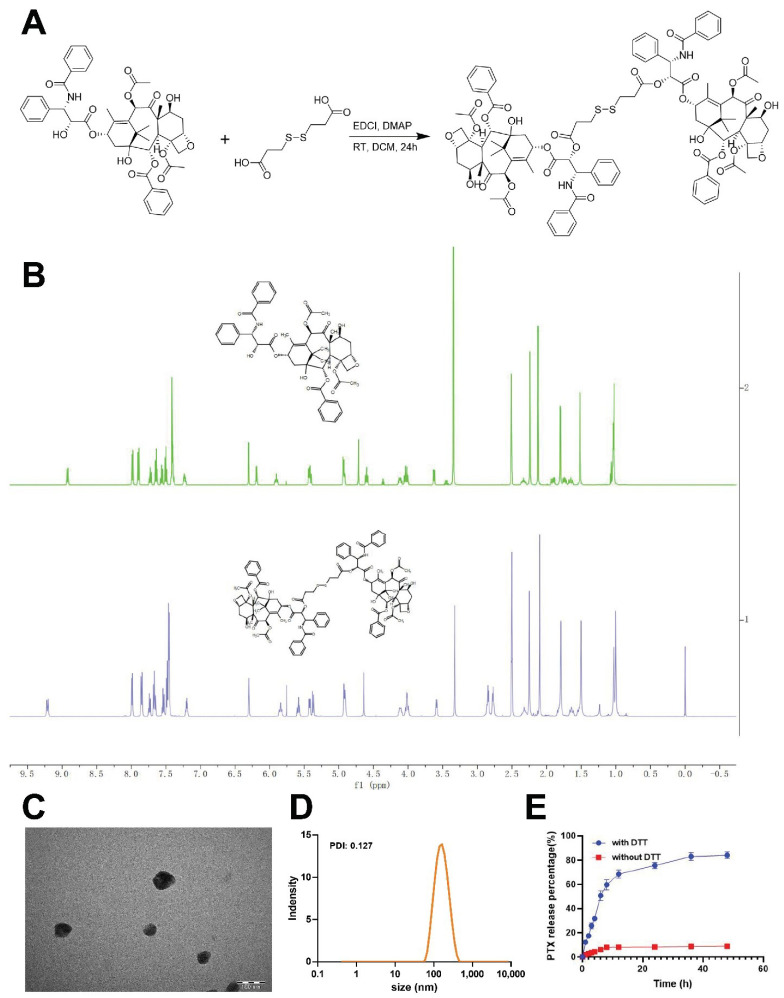
PTX-SS-PTX synthesis and characterization of TPG NPs. (**A**) The schematic of PTX-SS-PTX synthesis; (**B**) FTIR analyzing results of PTX and PTX-SS-PTX; TEM image (**C**) and dynamic light scattering (**D**) of TPG NPs (scale bar = 100 nm); (**E**) PTX release from TPG NPs in with/without DTT solutions (10 mM).

In this study, we attempted to combine prodrug technology and photodynamic therapy for more effective combination therapy. The combination of PTX and GEM has been reported to work synergistically in the treatment of pancreatic cancer. THPP has been added to acquire diagnostic and therapeutic properties to TPG nanoparticles, not only as a means of detection for drug delivery, but also to allow simultaneous photodynamic therapy. We first prepared PTX as a prodrug of PTX-S-S-PTX (Figure 1A) by using a disulfide linker. An ^1^H NMR spectral analysis showed that, compared with PTX, PTX-S–S-PTX presented two new proton peaks at δ 2.90 ppm; these peaks are methylene peaks (-CH_2_-CH_2_-S-S) from a disulfide linker (Figure 1B). These results indicated the structural correctness of the PTX-SS-PTX prodrug molecule. TPG nanoparticles were subsequently prepared using film hydration by dissolution of the PTX prodrugs, GEM, THPP, and DSPE-PEG in ethanol. Since the best tumor cell growth suppression was achieved at 50% PTX, we selected this ratio as the optimal ratio for subsequent experiments and mixed PTX with GEM in equal proportions to construct TPG NPs (Figure S1). The DLS and TEM results show that the TPG nanoparticles have a circular morphology and a uniform hydrodynamic size of 135 ± 9.7 nm (Figure 1C,D). The release of paclitaxel from the nanoparticles was significantly increased under the high reducing condition in vitro. This was demonstrated by the rapid release of PTX within the first few hours when the TPG nanoparticles were under reducing conditions, and the accumulation of released PTX reached approximately 68.5 ± 3.3% at 12 h. Thereafter, their release rates became very slow, whereas the control group without DTT showed very low drug release (Figure 1E).

### 3.2. TPG NPs Cellular Uptake

**Figure 2:**
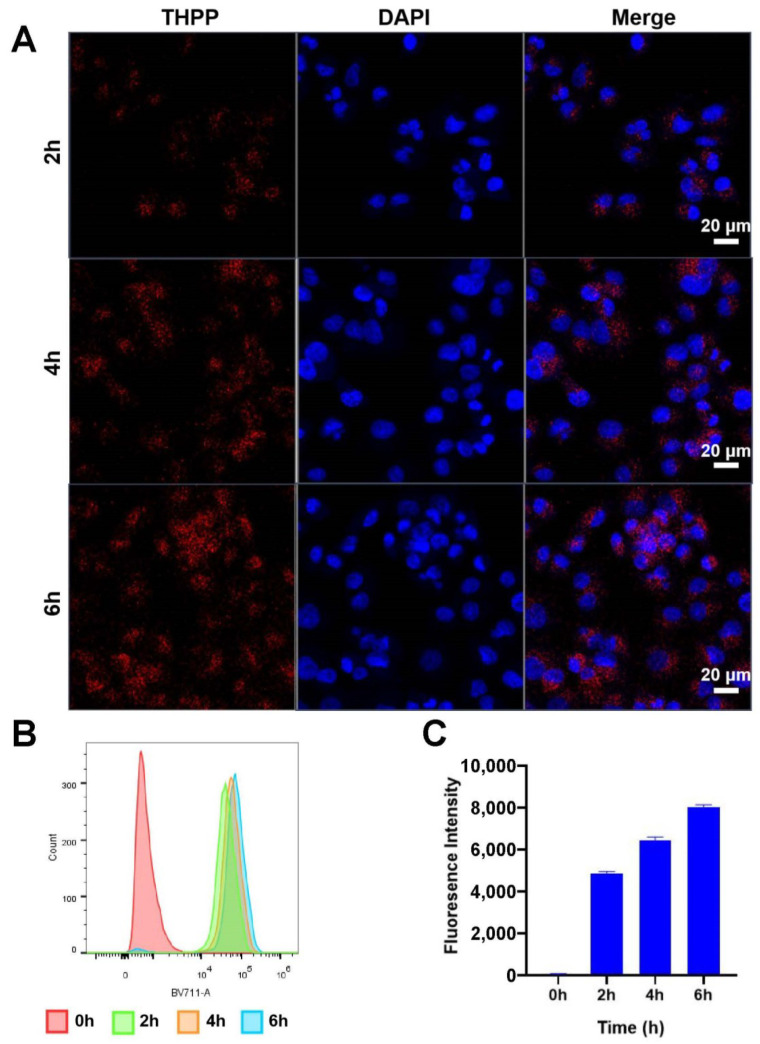
TPG NPs cellular uptake efficiency of PANC-1 cells. (**A**) Confocal microscopy images of PANC-1 cells after cells were incubated with TPG NPs for 2–6 h (scale bar = 20 μm; red: THPP; blue: DAPI); Flow cytometry analyzing result (**B**) and the quantitative data (**C**) of THPP fluorescence signal positive PANC-1 cells after being co-incubated with TPG NPs for 2–6 h.

Next, we assessed the TPG NPs uptake efficiency of pancreatic cancer PANC-1 cells by confocal microscope fluorescence imaging and flow cytometry analyzing. As shown in Figure 2A, after incubation of PANC-1 cells with TPG NPs for 2 h, a relatively pronounced red fluorescence signal of THPP was observed in the cytoplasm of PANC-1 cells, indicating that TPG NPs entered into the cells. Moreover, the intensity of the red fluorescence representing THPP increased at 4 h and 6 h, suggesting the increased uptake of TPG NPs by PANC-1 cells. Similarly, flow cytometry demonstrated the same cellular uptake trend as confocal microscope fluorescence imaging (Figure 2B,C). During 0–6 h, the uptake of TPG NPs by PANC-1 cells was increased in a time-dependent manner. All of the above results confirmed that PANC-1 cells have an excellent uptake of TPG NPs.

### 3.3. TPG NPs In Vitro Cytotoxicity

**Figure 3:**
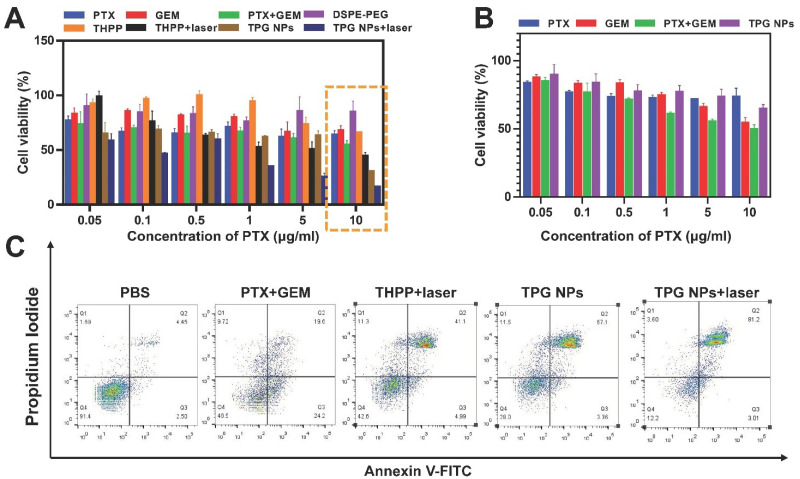
TPG NPs in vitro cytotoxicity assays. The cell viability of PANC-1 (**A**) and MCF-10A (**B**) cells tested by WST 1 assays. Cells were treated with different formulations for 48 h. (**C**) Flow cytometry analyzing of PANC-1 cells; cells were treated with different formulations for 48 h and stained with PI and Annexin V.

To evaluate the cytotoxicity of our prepared TPG NPs, we performed a standard WST-1 assay with pancreatic cancer PANC-1 and normal breast tissue MCF-10A cells. The cells were treated with different conditions (PTX, GEM, PTX + GEM, DSPE-PEG, THPP and TPG NPs) for 48 h, and selectively treated with a 650 nm laser (0.4 W/m^2^, 5 min). As shown in Figure 3A,B, the cell viability of PANC-1 and MCF-10A cells were all inhibited by a combination of PTX and GEM. And the TPG NPs exhibited similar PANC-1 cell viability inhibition ability as PTX + GEM when the PTX concentration was lower than 10 μg/mL, while high concentration (10 μg/mL) of TPG NPs could significantly decrease PANC-1 cell viability. Compared to TPG NPs only treated groups, the cell viability of PANC-1 cells treated with TPG NPs + laser was significantly decreased, and reached to the lowest of 16.3 ± 0.5% especially in the highest concentration of TPG NPs + laser (10 μg/mL PTX) treated group. These results indicated that TPG NPs have exacerbated the sensitivity of pancreatic cancer cells to photodynamic therapy and chemotherapy. This also indicated that both THPP and DSPE-PEG had virtually no cytotoxicity. Compared to the result in MCF-10A cells (Figure 3B), the cell viability was only decreased to 67 ± 3.6% by TPG NPs (10 μg/mL PTX), while the cell viability was decreased to 30 ± 1.6% in PANC-1 cells. The result demonstrated that TPG NPs was of a much lower lethality in MCF-10A cells, indicating the excellent biological histocompatibility of TPG NPs. Passive targeting induced by DSPE-PEG coating may be responsible for the different inhibition of TPG NPs on PANC-1 and MCF-10A cells proliferation. Otherwise, increased PTX release due to the reducing environment of PANC-1 cells was also one of the main reasons for selectively cell viability inhibition.

Both photodynamic reaction and chemotherapeutics (PTX, GEM) mainly induce cancer cell apoptosis to achieve therapeutic efficacy [26,27,28]. Therefore, we further assessed PANC-1 cells apoptosis by flow cytometry after different treatments for 48 h. As the result in Figure 3C, the proportion of late apoptotic cells in PBS group was 4.45%, while it was increased to 19.6% in the PTX + GEM group, and the proportion was surprisingly reached to 57.1% in the TPG NPs group. The most obvious late apoptosis (81.2%) was discovered in the TPG NPs + laser group, reflecting that our combined treatment achieved the best efficacy.

### 3.4. Lysosomal Escape of TPG NPs

**Figure 4:**
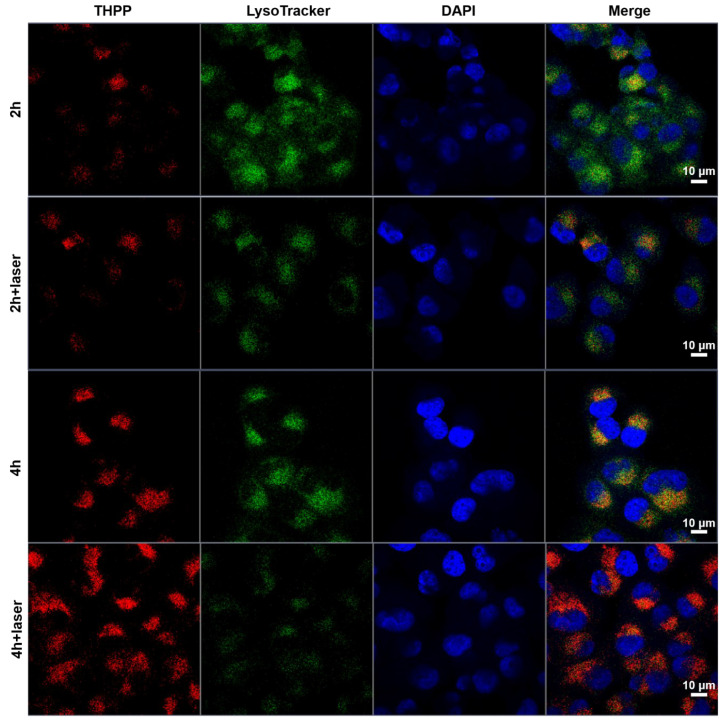
Lysosomal escape of TPG NPs. Confocal microscopy images of PANC-1 cells after treatment with TPG NPs for 2–4 h (with/without laser). Cells were stained with LysoTracker (**green**) and DAPI (**blue**) (**red**: THPP; scale bar = 10 μm).

Lysosomal entrapment is a major obstacle to be tackled in the drug delivery process, and efficient lysosomal escape is a major capability required for intracellular drug delivery vehicles [29]. Since laser irradiation of THPP could generate singlet oxygen, this course of action can destroy lysosomes and promote nanoparticle escape. Furthermore, we have also shown that this combined with 650 nm laser treatment can significantly increase TPG NPs-induced PANC-1 cell death. Therefore, to determine whether laser irradiation could facilitate lysosomal escape of TPG NPs in PANC-1 cells, we treated PANC-1 cells with TPG NPs for 2–4 h and selectively treated cells with 0.4 W/cm^2^ 650 nm laser for 5 min at the end of each time point. Next, a green fluorescent lysosomal dye was added to track lysosome. As the fluorescent imaging result in Figure 4 and Supplementary Figure S4 show, the red fluorescence representing THPP was co-localized with the green fluorescence representing the lysosome after the TPG NPs incubated with PANC-1 cells for 2 h and 4 h. However, laser irradiation significantly reduced the red-green fluorescence overlap in both 2 h and 4 h of TPG NPs treated groups, suggesting that laser irradiation allowed for the better escape of TPG NPs from the lysosomes. This result can be attributed to photodynamic reactions leading to membrane disruption, which further promoted the lysosomal escape of the drugs.

### 3.5. ROS Generation in PANC-1 Cells

**Figure 5:**
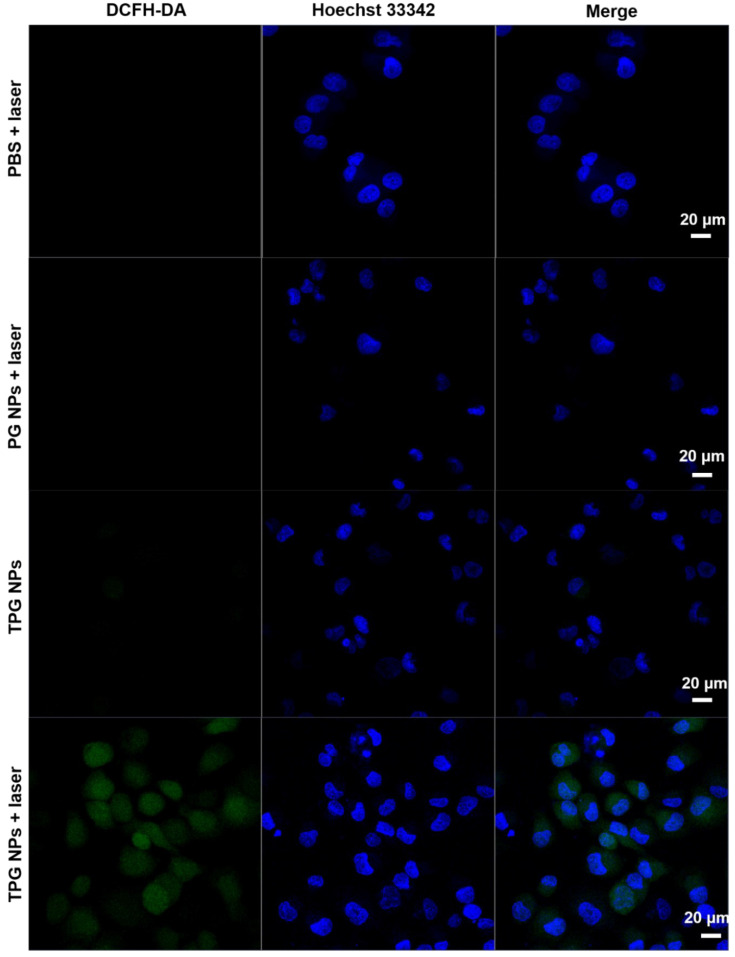
ROS generation in Panc-1 cells. Confocal microscopy images of PANC-1 cells after treatment with TPG NPs, PG NPs (NPs without THPP loading) and PBS for 24 h with/without laser irradiation and stained with DCFH-DA (**green**) and Hoechst (**blue**) (scale bar = 20 μm).

As we have described, TPG NPs combined with 650 nm laser treatment can significantly increase the cell apoptosis (Figure 3C) and decrease the cell viability (Figure 3A) compared to TPG NPs only treatment in PANC-1 cells. To investigate whether the increased cell death by TPG NPs + laser treatment was induced by THPP generated photodynamic reactions under 650 nm laser irradiation, we further detected the ROS level, which is the main product of photodynamic reactions, by monitoring a green fluorescent molecule DCF. The extra added DCFH-DA can freely cross the cell membrane and is hydrolyzed by intracellular esterase to produce DCFH, which accumulates in the cells. DCFH is oxidized by intracellular ROS to produce green fluorescent DCF, which is clearly visible under fluorescence microscopy. As the result in Figure 5 shows, the green fluorescent signal was obviously observed in TPG NPs + laser treated PANC-1 cells. However, no detectable fluorescent signal can be observed in both TPG NPs only and PT NPs (without THPP loading) or PBS + laser treated PANC-1 cells. The results demonstrated that TPG NPs combined with 650 nm laser treatment leaded better therapeutic results which was mainly attribute to photodynamic reactions generated more pronounced ROS to ablate cancer cells.

### 3.6. Biodistribution of TPG NPs In Vivo

**Figure 6:**
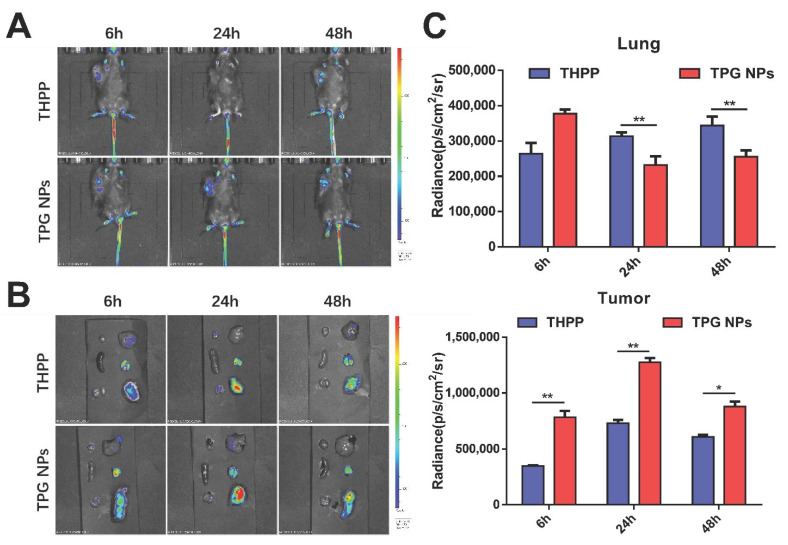
Biodistribution of TPG NPs in vivo. IVIS images (**A**,**B**) and the quantitative data (**C**) of C57BL/6 mice treated with different formulations for 6–48 h (Data represent the mean ± SD of three independent experiments. * *p* < 0.05, ** *p* < 0.01). Images were captured by monitoring the THPP signal, and the organs in (**B**) were arranged as heart and liver (first line), spleen and lung (second line), and kidney and tumor (third line).

Subsequently, to determine whether TPG NPs can be targeted to the tumor tissue and to observe its biodistribution, an in vivo fluorescence imaging assay was performed with tumor burden C57 BL/6 mice after 6, 24, and 48 h injection of TPG NPs from the tail vein. As shown in Figure 6A, compared with the THPP injected group, the TPG NPs injected group increased their NPs accumulation at the tumor site, and this was most pronounced at 24 h. Further removal of the tumors from the mice with normal tissue and fluorescence imaging also showed that the TPG NPs had the strongest fluorescence intensity in the tumors, and no significant accumulation in the normal tissue (Figure 6B,C). These results suggested that TPG NPs can also promote the targeted accumulation of drugs at tumor sites and improve the efficiency and specificity of drug utilization.

### 3.7. Anti-Tumor Activity and Biosafety In Vivo

**Figure 7:**
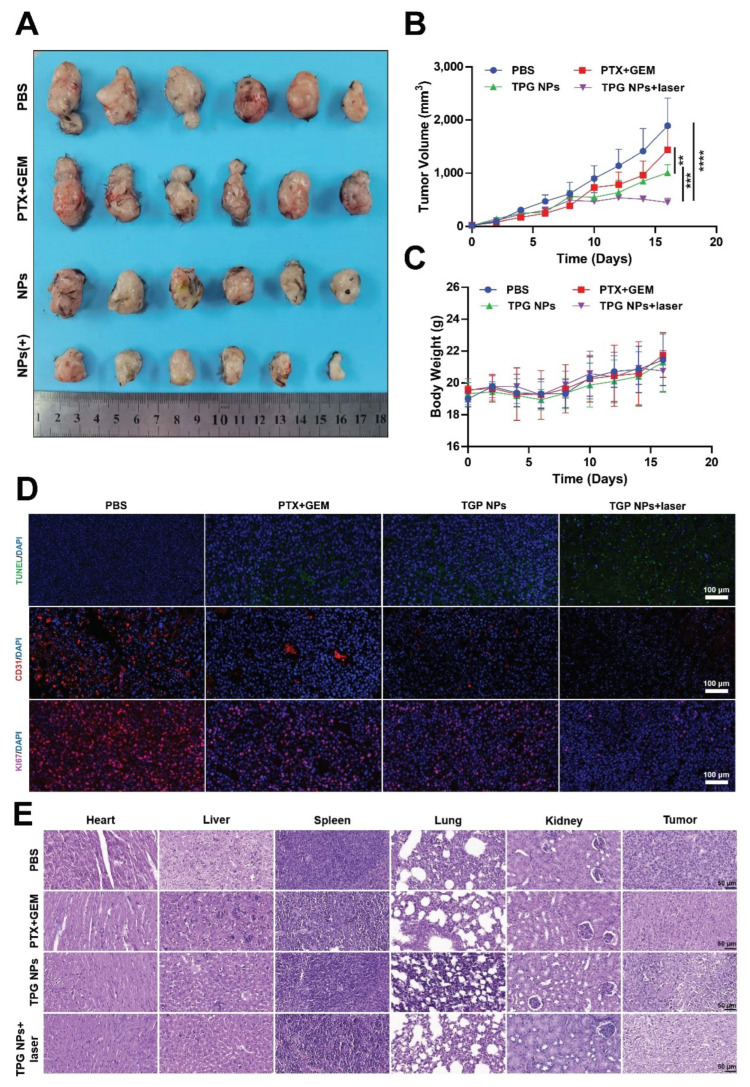
In vivo anti-tumor therapy and biosafety of TPG NPs. Image of tumors (**A**), recorded tumor growth (**B**) and body weight (**C**) of C57BL/6 mice treated with different formulations for 9 days. Date was expressed as the mean ± SEM (*n* = 6). ** *p* < 0.01. *** *p* < 0.001. **** *p* < 0.0001. (**D**) Fluorescence microscopy images of sliced tumor tissues stained with Tunel (green), CD31 (red), Ki67 (purple) and DAPI (blue) (scale bar = 100 μm). (**E**) Representative H&E-stained tumor slice images of mice after various treatment (scale bar = 50 μm).

Next, we further tested the in vivo anti-tumor activity of TPG NPs combined with 650 nm laser treatment in Panc02 cells formed pancreatic cancer C57BL/6 mice model. The mice were randomly divided into the following groups (*n* ≥ 6): (1) PBS (100 μL); (2) PTX (2.5 mg/kg) + GEM (2.5 mg/kg); (3) TPG NPs (equal to 2.5 mg/kg PTX); (4) TPG NPs + laser (650 nm, 0.5 W/cm^2^, 5 min). Considering that the most obvious TPG NPs accumulation at the tumor site was after 24 h injection, and laser irradiation was added the next day of each nanoformulations injection. It can be intuitively observed that the TPG NPs + the laser treated group had the best tumor suppression effect, followed by the TPG NPs group, but both were superior to the PTX + GEM pure drug treated groups from the representative photos of the isolated tumor tissue (Figure 7A). Meanwhile, the tumor volume curve was consistent with the result shown in the photos, and the combination therapy had the best efficacy (Figure 7B). It is not difficult to see from the body weight change curve of the mice that the TPG NPs + laser group showed little change in body weight compared to the control group (Figure 7C), indicating that the TPG NPs + laser treatment had limited toxic effects on the mice.

To further evaluate the anti-tumor activity in vivo, we performed an immunohistochemistry assay with tumor tissues, which were obtained from sacrificed mice to test three key markers that related to tumor growth. As the result shown in Figure 7D, the green fluorescent signal represented TUNEL, which is an apoptotic related biomarker was significantly increased in PTX + GEM, TPG NPs and TPG NPs + laser treated mice tumor tissues. The purple fluorescent signal representing the proliferation associated biomarker Ki67 was obviously decreased in PTX + GEM, TPG NPs and TPG NPs + laser treated mice tumors, especially in TPG NPs + laser treated groups. Moreover, we also detected the CD31 level of the tumor tissues with a red fluorescent dye labeled primary antibody. CD31 is a platelet endothelial cell adhesion molecule that is used to assess tumor angiogenesis. Compared with the PBS group, the staining results showed that CD31 fluorescence intensity was reduced in the PTX + GEM and TPG NPs groups, and the TPG NPs + laser group showed the lowest CD31 fluorescence intensity. All of these results demonstrated that TPG NPs can efficiently inhibit the tumor growth in vivo, and a better inhibition effect can be achieved by a combination of 650 nm laser irradiation. Otherwise, the treatment of TPG NPs, especially when combined with 650 nm laser irradiation, can significantly inhibit the vessel formation in the tumor tissue, which is a vital factor to tumor growth.

Finally, to determine the toxic side effects of TPG NPs on tissues, four groups (PBS; PTX + GEM; TPG NPs; TPG NPs + laser) of mice underwent a histological evaluation. The results showed that none of the treatments significantly damaged the normal organs (heart, liver, spleen, lung, kidney) of the mice, while the TPG NPs + laser group showed the strongest level of tumor cell nucleus ablation (Figure 7D). The above results demonstrated that TPG NPs + laser presented excellent therapeutic efficacy with a good biosafety profile. The combination of TPG NPs and PDT may thus serve as a promising candidate for pancreatic cancer treatment.

## 4. Discussion and Conclusions

Compared with single chemotherapy, combined chemotherapy with multiple drugs cannot only overcome the drug resistance of cancer, but can also improve the treatment effect [30]. However, the side effects of chemotherapy drugs remain a big problem. In this work, we synthesized a disulfide-bonded paclitaxel (PTX)-based prodrug (Figure 1A,B), which is in its nontoxic form but can be catalyzed into a cytotoxic form in reactive oxygen species (ROX) and glutathione (GSH) rich tumor tissue [31]. Thus, our designed PTX prodrug successfully avoided the premature release of PTX and reduced the cytotoxicity of TPG NPs against normal breast epithelial cell MCF-10A cells (Figure 3B) and organs (Figure 7E). 

**Scheme 1:**
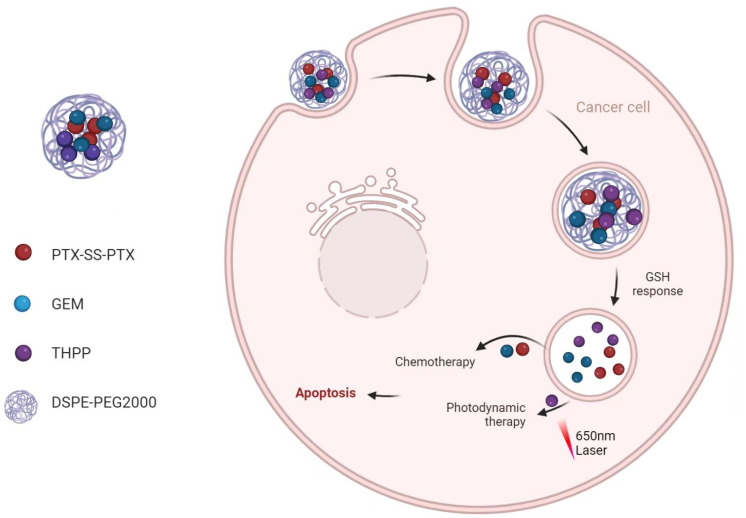
Schematic representation of TPG NPs preparation and drug release inside a pancreatic cancer cell.

Nevertheless, although the prodrug and nanotechnology has significant advantages (such as reduced side effect, enhanced stability, improved anti-tumor activity, etc.) for chemotherapy, the lack of long-term circulation ability of prodrug and prodrug based self-assembled nanoparticles will also bring great metabolic burden to the body [32]. Therefore, wrapping the PTX prodrug, THPP, and GEM with DSPE-PEG polymer (Scheme 1) came to be a good strategy. This is because the FDA approved DSPE-PEG polymer can not only keep the stability of TPG NPs, but also prolong the circulation time in the blood and resulted increased TPG NPs accumulation in tumor tissue (Figure 6) [33].

A lysosomal trap has always been considered as one of the most challenging problems that leads to failed drug delivery, and several efforts have been made to help nanoformulations escape from the lysosome [34]. An example would be the breaking of the membrane of lysosome by the cationic polymer owing to the proton sponge mechanism, and also by the fusion of liposomes with the lysosomal membrane. However, these methods also increased the cytotoxicity because of unexpected cell membrane destruction. In our work, we co-loaded the photosensitizer THPP in TPG NPs; it can be excited by 650 nm laser irradiation thus produce lysosomal membrane destructive ROS. Therefore, the chemo drugs PTX and GEM were better released in to the cytoplasm and exhibited enhanced antitumor activity both in vivo and in vitro under 650 nm laser irradiation conditions (Figure 3 and Figure 7). Moreover, the results of the in vivo experiments and also in vitro experiments sufficiently demonstrated that TPG NPs in the combination with a 650 nm laser can lead to a significant synergistic anti-tumor effect, proving the potential prospects of combined therapy of multi-chemotherapeutics and PDT, which provided a good candidate for the future combined therapy of pancreatic cancer. 

In summary, we designed a nanoplatform that can optimize the combined application of PDT and chemotherapy for pancreatic cancer-targeted therapy. Laser irradiation combined with TPG NPs, which formed by encapsulating the PTX prodrug, GEM, and photosensitizer THPP with DSPE-PEG, achieved a safe and efficient treatment.

## Data Availability

Not applicable.

## Supplementary Materials

The following supporting information can be downloaded at: https://www.mdpi.com/article/10.3390/pharmaceutics14112280/s1. Figure S1: Cell viability of PANC1 cells treated with different amount of PTX and GEM; Figure S2: Quantitative data of apoptotic cells (Data represent the mean ± SD of three independent experiments. * *p* < 0.05, ** *p* < 0.01, *** *p* < 0.001); Figure S3: NMR results of prodrug PTX-SS-PTX; Figure S4: Lysosomal escape of TPG NPs, colocalized with lysosome trackers.
