# Supplementary material for: Co-Delivery of Paclitaxel Prodrug, Gemcitabine and Porphine by Micelles for Pancreatic Cancer Treatment via Chemo-Photodynamic Combination Therapy
Source: Pharmaceutics. 2022 Oct 25;14(11):2280. doi: 10.3390/pharmaceutics14112280 (PMC9698062; doi:10.3390/pharmaceutics14112280)
Supplement: Supplementary file 1 [file pharmaceutics-14-02280-s001.zip › pharmaceutics-1929619-supplementary.pdf]

Supporting Information

Co-Delivery of Paclitaxel Prodrug, Gemcitabine and Porphine by Micelles for Pancreatic Cancer Treatment via Chemo-Photodynamic Combination Therapy

Qiwei Wu [1,2,†], Xiaodong Ma [1,3,4,†], Wenhui Zhou [3,4,5,†], Rong Yu [1], Jessica M. Rosenholm [3], Weizhong Tian [2], Lirong Zhang [1,*], Dongqing Wang [1,*] and Hongbo Zhang [3,4,*]

[1] Department of Radiology, Affiliated Hospital of Jiangsu University, Zhenjiang 212001, China
[2] Department of Medical Imaging, Affiliated Taizhou People's Hospital of Nanjing Medical University,
Taizhou 225300, China
[3] Pharmaceutical Sciences Laboratory, Faculty of Science and Engineering, Åbo Akademi University,
20520 Turku, Finland
[4] Turku Bioscience Centre, University of Turku and Åbo Akademi University, 20520 Turku, Finland
[5] Southern Medical University Affiliated Fengxian Hospital, Shanghai 201499, China
* Correspondence: tianchen861@ujs.edu.cn (L.Z.); wangdongqing71@ujs.edu.cn (D.W.); hongbo.zhang@abo.fi (H.Z.)
† These authors contribute equally to this work.

Cell lines and cell culture

PANC-1 and MCF-10A cell lines were obtained from American Type Culture Collection (ATCC, Manassas, VA, USA) and cultured in a constant humidity incubator containing 5 % CO_2 at 37 °C. PANC-1 cell line was cultured in DMEM medium (LONZA) supplemented with 10 % fetal bovine serum (FBS), penicillin (100 U/ml) and streptomycin (0.1 mg/ml). MCF-10A cell line was cultured in DMEM/F-12 (1:1) medium (LONZA) supplemented with 10% horse serum, 20 ng/ml epidermal growth factor, 0.5 μg/mL hydrocortisone, 10 μg/mL insulin, 1% NEAA, penicillin (100 U/ml) and streptomycin (0.1 mg/ml).

1. Synthesis and characterization of PTX-S-S-PTX

Briefly, PTX (2.0 g), DTDP (295.47 mg), EDCI (268.51 mg) and DMAP (171.68 mg) were dissolved in 30 mL of dichloromethane. The mixture was stirred at room temperature overnight. The reaction solution was extracted with water and dichloromethane, the catalyst was washed away, and the organic solution was dried with anhydrous magnesium sulfate. Finally, the crude product was purified by silica gel column chromatography to give the pure intermediate (yield = 78%), and characterized by a Bruker Daltonics microTOF-Q mass spectrometer (Bruker, Billerica, MA, USA).

2. In vitro cell viability assay

The PANC-1 and MCF-10A cells were seeded in 96-well plates (5×10^3 cells per well) and cultured at 37 °C, 5 % CO_2 overnight. Then the cells were exposed to different conditions (PTX, GEM, PTX+GEM, DSPE-PEG, THPP, THPP + laser, TPG NPs, TPG NPs + laser) for 72h, and then detected the cell viability by WST-1 (Dojindo, #150849-52-8) assay. The 650 nm laser was added with the power of 0.4 W/cm^2 for 5 min at 48 hours, and WST-1 was used according to the instructions of the reagent manufacturer. After adding WST-1 detection reagent, the plate was incubated in the dark for about 1-2 h at 37 °C. Absorbance (OD value) was measured at 450 nm wavelengths with microplate reader (Biotek, #Epoch2).

3. Statistical Analysis

All data were presented as the mean ± standard error of the mean (SEM). Statistical analysis was performed using Prism 7.0 software. The differences between groups were analyzed using Student's t test, one-way analysis of variance (ANOVA) or two-way ANOVA. $P<0.05$ was considered to statistically significant difference. All experiments biology repeated at least three times and all experiments were repeated at least two times.

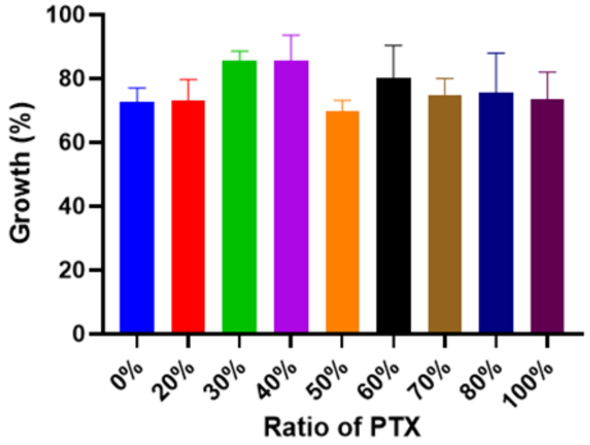

Figure S1. Cell viability of PANC1 cells treated with different amount of PTX and GEM.

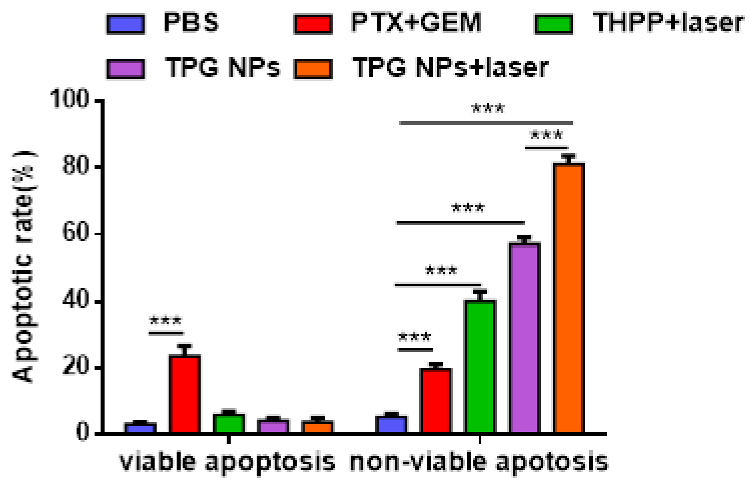

Figure S2. Quantitative data of apoptotic cells. （Data represent the mean ± SD of three independent experiments. * $p < 0.05$, ** $p < 0.01$, *** $p < 0.001$）.

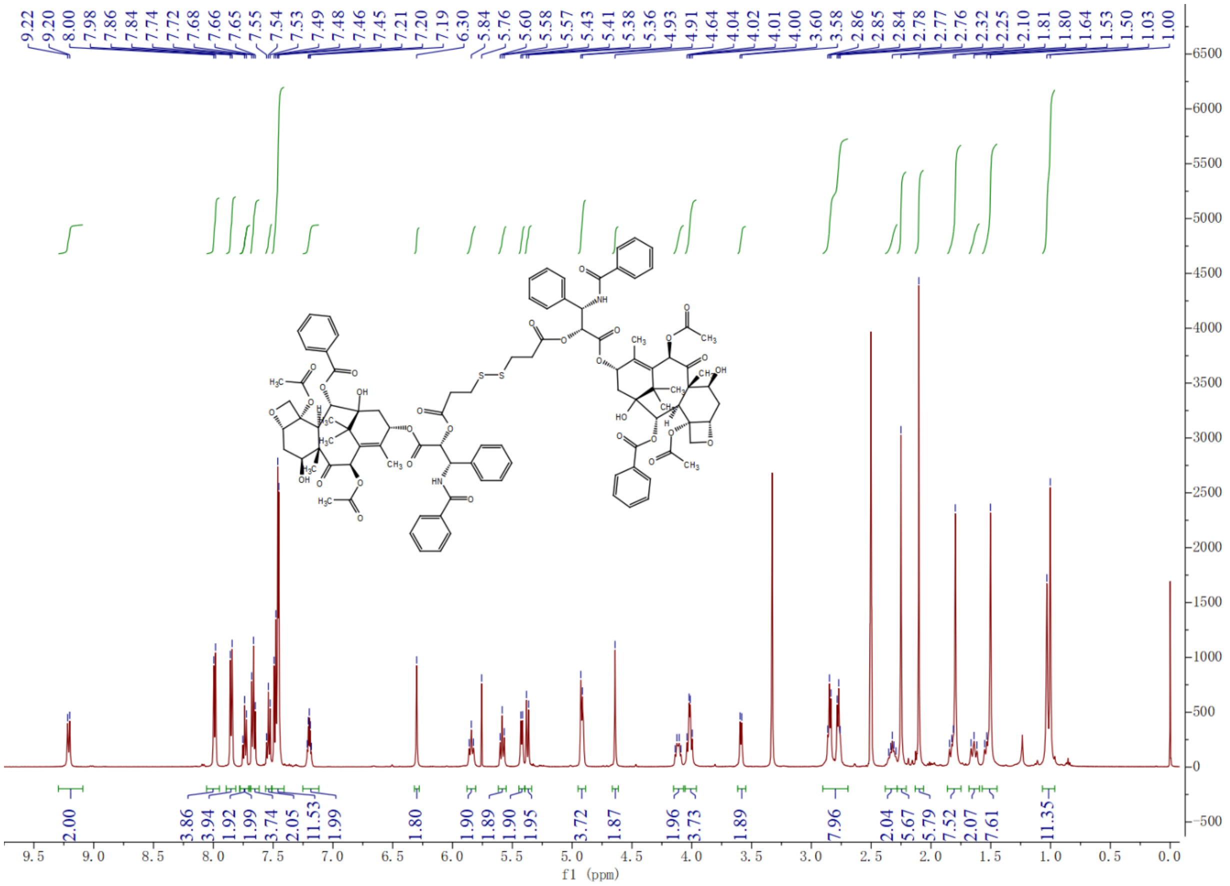

Figure S3. NMR results of prodrug PTX-SS-PTX.

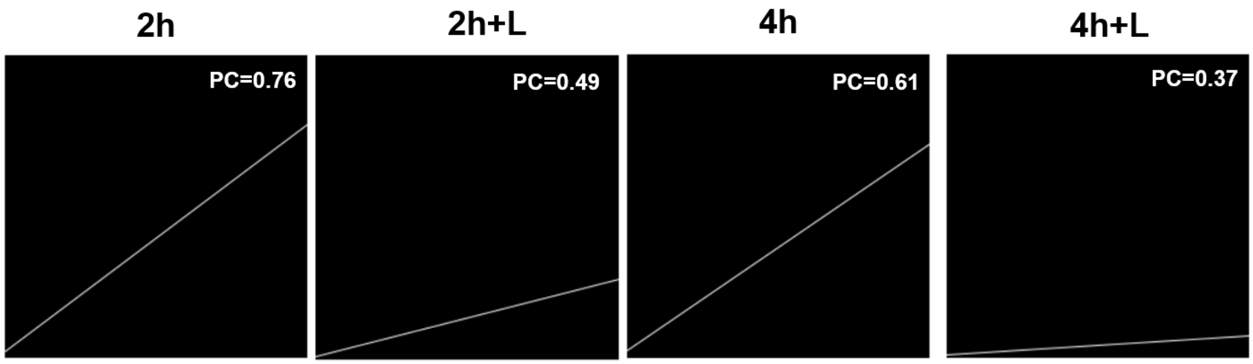

Figure S4. Lysosomal escape of TPG NPs, colocalized with lysosome trackers**.**
